# Identification of scintillation signatures on GPS signals originating from plasma structures detected with EISCAT incoherent scatter radar along the same line of sight

**DOI:** 10.1002/2016JA023271

**Published:** 2017-01-13

**Authors:** Biagio Forte, Chris Coleman, Susan Skone, Ingemar Häggström, Cathryn Mitchell, Federico Da Dalt, Tommaso Panicciari, Joe Kinrade, Gary Bust

**Affiliations:** ^1^ Department of Electronic and Electrical EngineeringUniversity of BathBathUK; ^2^ Electrical and Electronic Engineering DepartmentUniversity of AdelaideAdelaideSouth AustraliaAustralia; ^3^ Schulich School of EngineeringUniversity of CalgaryCalgaryAlbertaCanada; ^4^ EISCAT Scientific AssociationKirunaSweden; ^5^ Department of PhysicsUniversity of LancasterLancasterUK; ^6^ Applied Physics LaboratoryThe Johns Hopkins UniversityBaltimoreMarylandUSA

**Keywords:** GPS, scintillation, incoherent scatter radar, EISCAT, auroral arcs, ionospheric trough

## Abstract

Ionospheric scintillation originates from the scattering of electromagnetic waves through spatial gradients in the plasma density distribution, drifting across a given propagation direction. Ionospheric scintillation represents a disruptive manifestation of adverse space weather conditions through degradation of the reliability and continuity of satellite telecommunication and navigation systems and services (e.g., European Geostationary Navigation Overlay Service, EGNOS). The purpose of the experiment presented here was to determine the contribution of auroral ionization structures to GPS scintillation. European Incoherent Scatter (EISCAT) measurements were obtained along the same line of sight of a given GPS satellite observed from Tromso and followed by means of the EISCAT UHF radar to causally identify plasma structures that give rise to scintillation on the co‐aligned GPS radio link. Large‐scale structures associated with the poleward edge of the ionospheric trough, with auroral arcs in the nightside auroral oval and with particle precipitation at the onset of a substorm were indeed identified as responsible for enhanced phase scintillation at L band. For the first time it was observed that the observed large‐scale structures did not cascade into smaller‐scale structures, leading to enhanced phase scintillation without amplitude scintillation. More measurements and theory are necessary to understand the mechanism responsible for the inhibition of large‐scale to small‐scale energy cascade and to reproduce the observations. This aspect is fundamental to model the scattering of radio waves propagating through these ionization structures. New insights from this experiment allow a better characterization of the impact that space weather can have on satellite telecommunications and navigation services.

## Key Points

- EISCAT incoherent scatter radar beam co‐aligned with GPS satellite line of sight
- Absence of large‐scale to small‐scale energy cascade in the auroral *E* and *F* regions
- Scintillation‐inducing plasma gradients in the auroral *E* and *F* regions

## 1 Introduction

The propagation of radio waves through drifting inhomogeneties in the spatial distribution of electron density may lead to degradation of the overall signal manifesting itself as fluctuations of the phase and amplitude components of the radio waves, a phenomenon known as scintillation [*Yeh and Liu*, 1982; *Aarons*, 1982; *Basu et al*., 1999; *Basu et al*., 2001; *Fremouw et al*., 1978]. Scintillation represents a serious threat to satellite telecommunication as well as satellite navigation systems as it can disrupt a service entirely, resulting in increased errors and outages [*Seo et al*., 2011; *Kintner et al*., 2007; *Skone et al.*, 2001; *Skone and de Jong*, 2000; *Skone*, 2001].

In the presence of scintillation the energy received at the antenna is lower than in the absence of scintillation, a consequence of the scattering by electron density inhomogeneities in the ionosphere. When radio waves scatter through electron density structures the wave energy is scattered away from the original propagation direction, leading to a lower signal being recorded at the receiving antenna.

The scattering of radio waves that leads to ionospheric scintillation is often classified into three distinct regimes: (a) weak scattering, (b) moderate‐to‐strong scattering, and (c) strong scattering [*Yeh and Liu*, 1982; *Booker and MajidiAhi*, 1981].

In the case of weak scattering the propagation problem can often be approximated by means of a single‐phase changing screen containing a distribution of phase changes (as a consequence of variations onto the spatial distribution of the refractive index) to be superimposed on the incident wave front [*Booker and MajidiAhi*, 1981; *Rino*, 1979]. In the case of moderate‐to‐strong scattering the use of multiple phase screens becomes necessary to accommodate larger and cumulative phase variations in response to propagation through electron density structures with greater extent [*Knepp*, 1983a; *Uscinski*, 1968; *Carrano et al*., 2011]. However, multiple phase screens should be placed with separations no more than the correlation length of the electron density structures to properly reproduce strong scattering (i.e., as opposed to the distance effect).

The correlation length of plasma structures that gives rise to strong and saturating scintillation is hard to establish. Previous studies at low latitudes demonstrated that strong scintillation occurs in the vicinity of plasma bubble walls as well as in the presence of plumes of ionization [*Valladares et al.*, 2004; *Rodrigues et al*., 2004; *Sripathi et al*., 2008; *Lee et al*., 2009; *Costa et al*., 2011; *Carrano et al*., 2012; *Patra et al*., 2014]. At high latitudes strong scintillation is caused by the presence of plasma patches and particle precipitation [*Skone et al*., 2001; *Mitchell et al*., 2005; *Forte*, 2005; *Smith et al*., 2008; *Prikryl et al*., 2010; *Kinrade et al*., 2012, 2013; *van der Meeren et al*., 2014, 2015]. In the case of satellite signals the propagation geometry is to be properly accounted for in the propagation problem together with the aspect ratio of electron density structures which signals need to traverse. In the case of GPS signals at high latitudes the propagation occurs at a significant angle to the magnetic field lines, along which plasma structures tend to elongate [*Forte and Radicella*, 2004].

The purpose of the experiment reported here was to provide insights into the type of structures that originate scintillation on GPS signals at auroral latitudes. In particular, the identification and characterization of plasma gradients responsible for certain levels of scintillation was attempted by maintaining the European Incoherent Scatter (EISCAT) UHF radar along the same line of sight as a given GPS satellite (the signal recorded by means of a GPS scintillation monitor co‐located with EISCAT UHF transmitter at Tromso). The measurements described here were also utilized in a companion paper [*Chartier et al*., 2016], which focussed on the modeling of the scintillation observations by means of a propagation model based on 3‐D modeling of the propagation medium according to EISCAT electron density profiles. Optimum values of ionizations structures (e.g., outer scale and axial ratios) which provided the best fit to the observed scintillation were identified [*Chartier et al*., 2016]. Here the identification and quantification of the contribution from *E* and *F* region irregularities to the observed L band scintillation is provided through the combination of EISCAT and GPS observations under different circumstances.

## 2 Data and Methodology

The measurement campaign took place in October 2013 (see Table 1 for details), when different GPS satellites were followed with the EISCAT UHF radar during these measurements. Here events from 16 October 2013 and 17 October 2013 only are reported.

**Table 1:** Summary of GPS Satellites Followed by the Radar Throughout the Measurement Campaign

Date	GPS Satellite Followed by the Radar	Time Interval (UT)
07 October 2013	PRN22	14:00–15:45
	PRN32	15:50–18:45
	PRN23	18:50–21:00
16 October 2013	PRN19	14:00–14:55
	PRN32	15:00–18:05
	PRN23	18:10–21:00
17 October 2013	PRN23	18:00–21:00
18 October 2013	PRN11	14:00–15:30
	PRN32	15:35–17:55
	PRN23	18:00–21:00

The positions of GPS satellites to be followed were determined in advance on the basis of the projection of the ephemeris in the future by using an SP3 file (http://igscb.jpl.nasa.gov/igscb/data/format/sp3_docu.txt) released the day before each of the days during the measurement campaign. Those positions were determined at 5 min intervals to cover the entire duration of the measurement [*Forte et al*., 2013].

**Figure 1:**
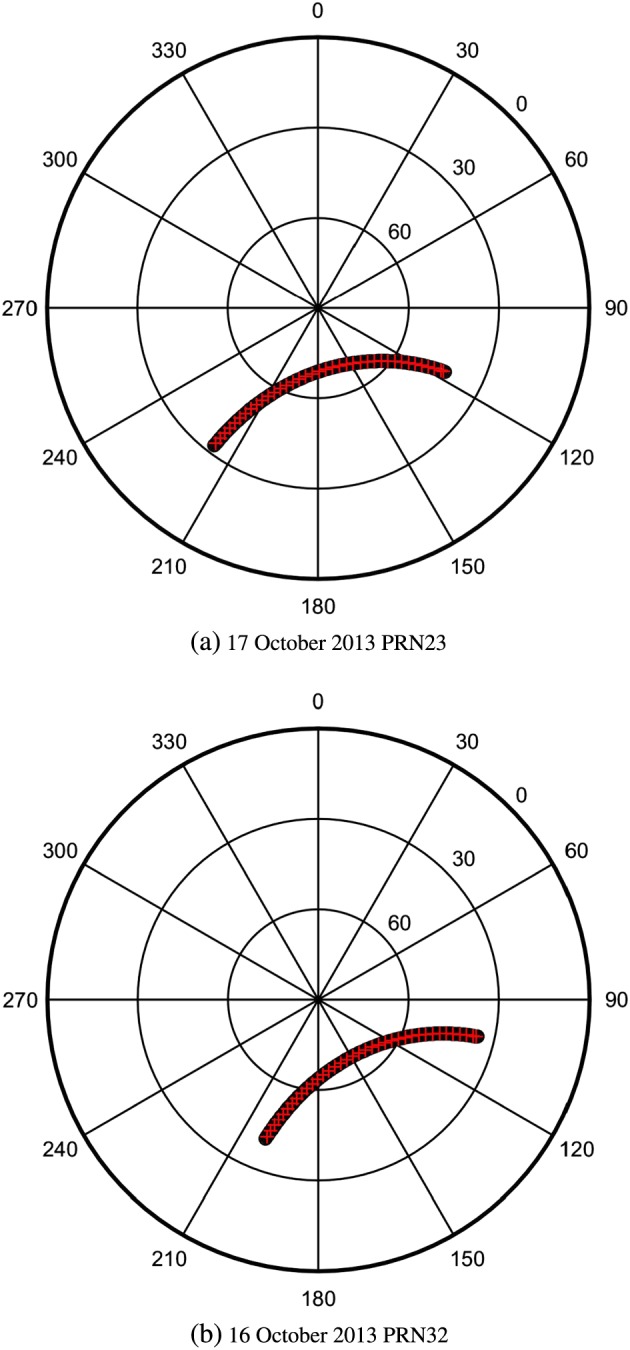
Trajectories of the GPS satellites (black circles) followed by the radar (red crosses) in terms of azimuth and elevation angles.

The EISCAT UHF radar was pointed toward the selected satellite by remaining fixed in a given position (defined in terms of azimuth and elevation) for 5 min, then repositioning into the new direction in the next interval, and so on (see Figure 1). At each fixed position the GPS satellite line of sight was moving and traversing the radar line of sight in each 5 min interval. During each 5 min interval the radar was measuring and collecting backscattered power which was then converted into electron density profiles by using the typical GUISDAP analysis toolbox (http://www.eiscat.com/groups/Documentation/UserGuides/GUISDAP/) [*Huuskonen and Lehtinen*, 1996]. EISCAT electron density profiles were subsequently calibrated following standard procedure (see *Forte et al*. [2013] for further details). The spatial resolution of the EISCAT radar measurements is of the order of 2 km in range.

Co‐located with the EISCAT radar was a Novatel Global Navigation Satellite Systems Ionospheric Scintillation and total electron content (TEC) Monitor (GPStation‐6; http://www.novatel.com/products/scintillation‐tec‐monitor/) capable of measuring ionospheric parameters (i.e., TEC, rate of change of TEC, and scintillation indices *σ*_*ϕ*_ and *S*_4_) at 1 min intervals as well as signals phase and amplitude components at 50 Hz rate [*Van Dierendonck et al*., 1993].

**Figure 2:**
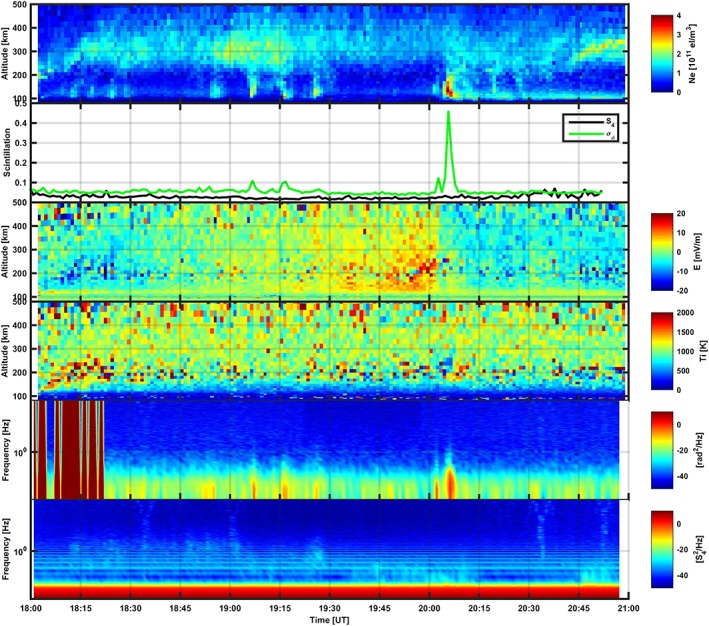
The event measured on 17 October 2013, characterized by (from top to bottom) electron density profiles, GPS scintillation indices, electric field, ion temperature, PSD for the detrended carrier phase, and PSD for the normalized intensity.

**Figure 3:**
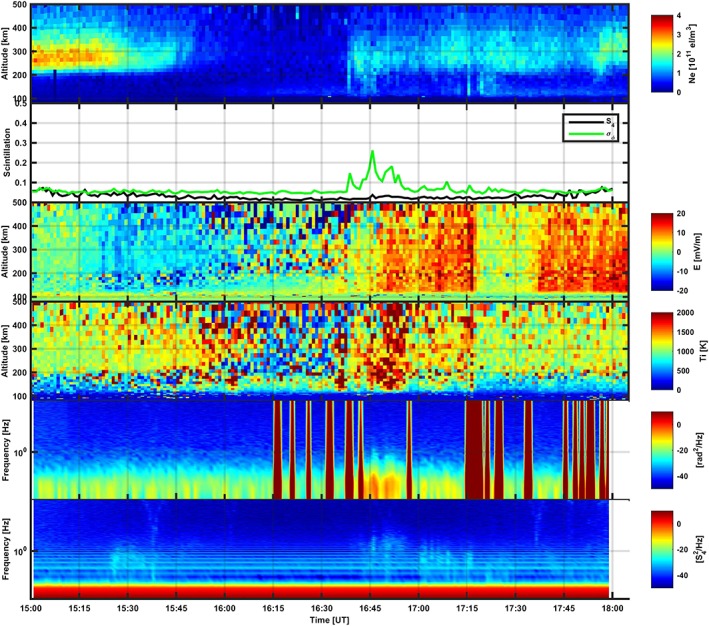
The event measured on 16 October 2013, characterized by (from top to bottom) electron density profiles, GPS scintillation indices, electric field, ion temperature, PSD for the detrended carrier phase, and PSD for the normalized intensity.

Figure 2 shows measurements for the event on 17 October 2013, while Figure 3 shows measurements for the event on 16 October 2013. Figures 2 and 3 show electron density altitude profiles measured by EISCAT; scintillation indices corresponding to the GPS satellites followed by the EISCAT UHF radar, measured by means of the GPS scintillation monitor along a co‐aligned direction; altitude profiles of the electric field calculated from the ion temperature and velocity following *Banks and Kockarts* [1973]; altitude profiles of the ion temperature; and power spectral densities (PSD) for detrended carrier phase and for the normalized intensity of the GPS signals. PSDs were calculated over 3000 values at time covering an entire minute interval at 50 Hz sampling rate. Red stripes along the phase PSDs originated because of glitches generated by the GPS monitor clock.

In this experiment, the integration time of 60 s was chosen for the comparison with GPS scintillation indices (estimated over a 60 s time interval) as well as to enhance sensitivity to smaller‐scale electron density structures responsible for scintillation.

An integration time of 60 s gave five different electron density profiles (one every 60 s) along the same direction. The five different 60 s profiles provided information on the temporal evolution (e.g., energy cascade, drift, and recombination) of the plasma structures detected (Figures 2 and 3).

An integration time of 60 s implies a maximum spatial distance, between the radar direction and the offset satellite raypath at the beginning and end of a given 5 min interval, of the order of 10 km in a direction transversal to the beam direction at about 200 km in altitude (and more at higher altitudes), assuming a GPS signal scan velocity of the order of 80 m/s at 200 km in altitude [*Kaplan and Hegarty*, 2006]. This provides an upper limit for the transversal spatial scales sampled by the GPS satellite signal, which together with electron density profiles provide information on the volume of ionization structures originating particular scintillation signatures.

**Figure 4:**
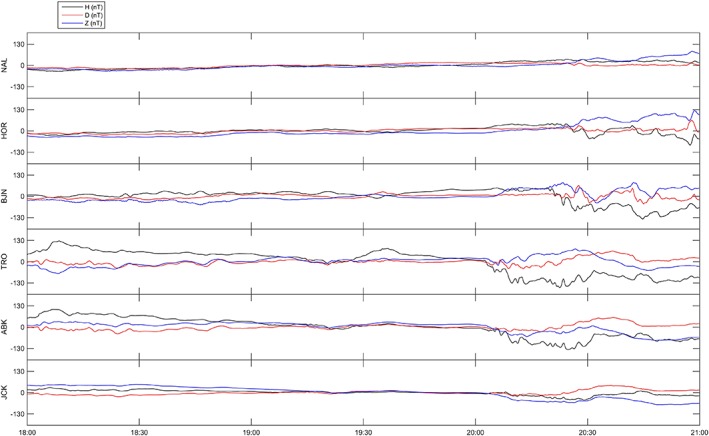
Magnetograms (variations in HDZ components in nT) from the IMAGE network (17 October 2013) from stations Ny Alesund (NAL), Hornsund (HOR), Bear Island (BJN), Tromso (TRO), Abisko (ABK), and Jackvik (JCK).

Components of the magnetic field are shown in Figures 4 (17 October 2013) and 5 (16 October 2013) for the magnetometers at Ny Alesund (NAL), Hornsund (HOR), Bear Island (BJN), Tromso (TRO), Abisko (ABK), and Jackvik (JCK), part of the International Monitor for Auroral Geomagnetic Effects (IMAGE) network [*Tanskanen*, 2009].

**Figure 5:**
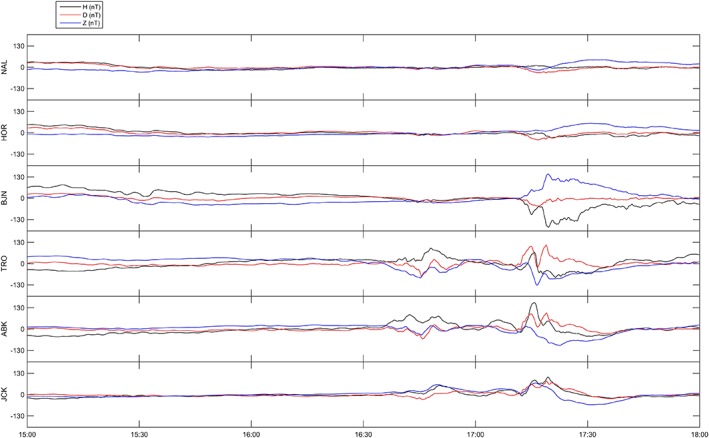
Magnetograms (variations in HDZ components in nT) from the IMAGE network (16 October 2013) from stations Ny Alesund (NAL), Hornsund (HOR), Bear Island (BJN), Tromso (TRO), Abisko (ABK), and Jackvik (JCK).

## 3 Results

### 3.1 17 October 2013 PRN23 18–21 UT

In the case of the measurements taken on 17 October 2013 (Figure 2), the EISCAT UHF radar detected several structures in the electron density profiles, some of which originating a corresponding signature on scintillation indices. Throughout the measurements, a peak in ionization was around 300 km in altitude, with values of the order of 2.5 × 10^11^ el m^− 3^ on average and higher values between 18:50 and 19:30 UT and between 20:45 and 21:00 UT.

Between 18:00 and 18:30 UT some structures in the electron density profiles were detected below 200 km in altitude (extending less than 100 km in altitude each) with values of 1.5 × 10^11^ el m^− 3^ on average. These structures did not cause any remarkable signatures neither on *σ*_*ϕ*_ nor *S*_4_, although a minor feature on *S*_4_ could be noticed at about 18:20 UT; no signatures could be identified on the intensity and phase PSDs (although phase PSDs were heavily masked by cycle slips in that time interval—Figure 2).

Between 18:30 and 19:00 UT another feature in the electron density profile (similar to those observed between 18:00 and 18:30) could be noticed (around 18:50 UT) which did not correspond to any signature on scintillation indices. The intensity PSDs did not show any modification while the phase PSDs showed an enhancement at lower temporal frequencies corresponding to the feature at 18:50 UT.

Between 19:00 and 19:30 UT three distinct electron density structures were detected with values of the order of 2.5 × 10^11^ el m^− 3^, extending between 100 km and 200 km in altitude and within 10 km along the apparent GPS satellite direction across the radar line of sight (assuming an apparent satellite raypath scan velocity of 80 m/s at 200 km of altitude) over a background ionization below 1 × 10^11^ el m^− 3^. Around 300 km in altitude, the electron density intensified to about 2.5 × 10^11^ el m^− 3^ as well. Enhancements on phase PSDs at lower temporal frequencies were noticed in correspondence to these electron density structures, although only the first two of them originated a signature on *σ*_*ϕ*_ (about 0.1 rad), leaving intensity PSDs and *S*_4_ unmodified. The spectral modifications noticed on the phase PSDs implied an enhancement at lower temporal frequencies and spectral broadening up to 0.6 Hz.

Between 19:30 and 20:00 UT no particular structures were detected by the radar and no particular signatures were observed either on the scintillation indices or on the PSDs.

Between 20:00 and 20:30 UT a first structure in electron density of about 2.5 × 10^11^ el m^− 3^ was detected followed by a more intense one of about 3.5 × 10^11^ el m^− 3^ on average, fading onto lower values afterward. A volume of ionization of about 3.5 × 10^11^ el m^− 3^ on average, extending between 100 km and 200 km in altitude and within 10 km along the apparent GPS satellite direction across the radar line of sight over a background ionization below 1 × 10^11^ el m^− 3^ was responsible for enhancing *σ*_*ϕ*_ up to 0.45 rad (Figure 2). Spectral modifications were observed on the phase PSDs, with enhancement at lower temporal frequencies and spectral broadening up to 1 Hz. The intensity PSDs showed some enhancement above the Fresnel frequency as well as an increase in the Fresnel temporal frequency. After the most intense structure a sporadic *E* layer (diffuse aurora) established itself until 21:00 UT. Some structures in the electron density profiles of about 1.5 × 10^11^ el m^− 3^ and extending about 100 km in altitude were detected without any corresponding signature on either scintillation indices or PSDs.

Between 20:30 and 21:00 UT a sporadic *E* layer persisted with an intensification of electron density between 300 and 350 km in altitude, with values of the order of 2.5 × 10^11^ el m^− 3^ developing over apparently narrower layers.

During the whole interval of measurements, Tromso was situated under the evening side of the auroral oval where auroral structures were frequently drifting. Changes in the electric field were observed as a consequence of polarization electric fields [*Aikio et al*., 1993; *de la Beaujardiere et al*., 1977; *Lanchester et al*., 1996; *Opgenoorth et al*., 1990]. The abrupt ionization enhancement detected between 20:00 and 20:30 UT coincided with a substorm onset (Figures 2 and 4) which was responsible for the associated particle precipitation. The timing of the features observed through co‐aligned radar and GPS measurements are consistent with signatures observed through magnetometer data [*Tanskanen*, 2009]. The classical negative H (N‐S) perturbation is indeed characteristic of auroral substorm onset at approximately 20:06 UT and associated energetic electron precipitation in the E region.

### 3.2 16 October 2013 PRN32 15–18 UT

In the case of the measurements taken on 16 October 2013 (Figure 3), the EISCAT UHF radar beam detected some structures in the electron density distribution, responsible for enhancements in the phase scintillation index. Throughout the measurements, the *F* region daytime peak in ionization was noticeable between 200 and 400 km in altitude, with values of the order of 3 × 10^11^ el m^− 3^ on average between 15:00 and 15:45 UT. Then, the radar beam traversed a gap in the F region electron density between 15:45 and 16:30 UT, due to the transition between dayside and nightside. Subsequently, more ionization in the *F* region was encountered with values of the order of 1.5 × 10^11^ el m^− 3^ until the end of that given satellite pass (at 18:05 UT). Moreover, a sporadic *E* layer was noticed after 16:00 UT.

Between 15:00 and 16:30 UT the radar transited from a daytime peak in the *F* region to the nightside in such a time interval. The transition from the *F* region daytime ionization peak into the gap in electron density between 15:45 and 16:30 UT did not originate any enhancement on *σ*_*ϕ*_ and *S*_4_ (Figure 3). No spectral modifications could be observed on both intensity and phase PSDs (Figure 3).

Between 16:30 and 17:30 UT the radar detected two distinct structures with values of the order of 1.5 × 10^11^ el m^− 3^, extending between 100 km and 200 km in altitude and within 10 km along the apparent GPS satellite direction across the radar line of sight over a background ionization below 1 × 10^11^ el m^− 3^. The first of these structures was detected between 16:40 and 16:50 UT, while the second occurred between 17:20 and 17:30 UT. The first structure did enhance *σ*_*ϕ*_ up to 0.3 rad in the same time interval, while the second structure caused a barely noticeable increase above the oscillator noise floor (i.e., 0.07 rad [*Van Dierendonck et al*., 1993]). Both these structures did not cause any appreciable signature on *S*_4_. Phase PSDs were contaminated by clock glitches during this particular interval of measurements. Nevertheless, spectral modifications could be appreciated on the phase PSDs, with enhancement at lower temporal frequencies and spectral broadening up to 1 Hz. The intensity PSDs showed some tenuous higher‐frequency enhancement above the Fresnel frequency in relation to these structures.

Between 17:30 and 18:05 UT the radar detected intensification in the *F* region electron density at about 18:00 UT, with values increasing up to 2.5 × 10^11^ el m^− 3^ in some cases, extending between 200 km and 400 km in range. Scintillation indices were unmodified during this time interval.

Between 16:30 and 17:00 UT the poleward edge of the ionospheric trough was passing over Tromso, as indicated by the typical magnetic positive bay on the magnetic horizontal H component. Changes in the electric field were observed in correspondence to the ionospheric trough as a consequence of ionospheric convection [*Moffett and Quegan*, 1983; *Roger et al*., 1992]. After 17:00 UT, Tromso was situated under the eveningside of the auroral oval where auroral structures were frequently drifting (Figures 3 and 5).

## 4 Discussion

Enhancements on the phase scintillation index *σ*_*ϕ*_ were observed, corresponding to large‐scale ionization structures present along the GPS signal propagation direction (and co‐aligned with the EISCAT UHF radar beam within the experiment accuracy). The presence of large‐scale ionization structures could be inferred from structures on the electron density profiles and corresponding enhancement of phase PSDs at lower temporal frequencies. In some cases, the enhancement was accompanied by spectral broadening up to 1 Hz (Figures 2 and 3). The large‐scale electron density structures introduced gradients in the refractive index distribution which in turn superimposed phase variations on the GPS signal propagating through. In general, the intensity scintillation index remained very small and constant throughout all of the measurements, and the PSDs of the normalized intensity did not show any distinct spectral modifications.

A possible explanation of these observations may be as follows. The low‐frequency spectral enhancement on the PSD of the detrended GPS carrier phase, together with enhancements in the phase scintillation index *σ*_*ϕ*_, originate from large‐scale auroral ionization structures in the *E* and *F* regions. However, these large‐scale ionization structures did not cascade into smaller‐scale structures (i.e., absence of enhanced amplitude scintillation). The inhibition of large‐ to small‐scale energy cascade led to enhancements in phase scintillation without any enhancement in amplitude scintillation, when a fixed cutoff frequency is utilized to detrend the carrier phase [*Forte and Radicella*, 2002]. The absence of an energy cascade to smaller scales could be due to higher recombination rates and/or strong conductivity along the field lines. Despite the fact that electron thermal diffusion can be a possible instability mechanism in the auroral *E* region plasma [*Shalimov and Haldoupis*, 1995], more measurements and theory are necessary to understand this issue and reproduce the observations. In particular, the observations would be reproduced by an instability mechanism that (a) operates in the *E* region of the ionosphere, (b) injects free energy at larger scales, and (c) does not allow for an energy cascade to smaller scales.

From the comparison between electron density profiles and co‐aligned scintillation indices it is evident that the scintillation was induced by ionization structures in both *E* and *F* regions. However, the causal relationship between ionization structures, and corresponding scintillation, is hard to explain on the basis of Figures 2 and 3 alone.

In order to identify and quantify the causal relationship between scintillation and ionization structures in the *E* and *F* regions, the model by *Booker and MajidiAhi* [1981] was invoked [*Vats et al.*, 1981; *Forte*, 2008, 2012]. This model enables the estimation of scintillation that develops during propagation through a thick medium characterized by a mean square fractional fluctuation of the ionization spatial density $\bar{{\left(\frac{\Delta N}{N}\right)}^{2}}$, a mean ionization density *N*, and a slab thickness *D*. The propagation through such an ionization layer introduces mean square phase fluctuations described by [*Booker*, 1981; *Booker and MajidiAhi*, 1981; *Vats et al*., 1981; *Forte*, 2008, 2012]:

(1) $$\bar{{\left(\Delta \phi \right)}^{2}}=4r_{e}^{2}N^{2}\bar{{\left(\frac{\Delta N}{N}\right)}^{2}}\lambda^{2}L_{0}D\,\sec\chi$$

where *r*_*e*_ is the classical electron radius, *L*_0_ is the outer scale, *D* is the layer thickness, and *λ* is the wavelength of the signal considered.

An expression similar to (1) was derived by *Knepp* [1983b] by assuming that the layer thickness is greater than the correlation length of the irregularities. Knepp assumed a three‐dimensional ionization power spectrum of the form *K* ^− 4^, with irregularities infinitely elongated in a direction orthogonal to the propagation direction. In this case, the structure function was approximated by a quadratic, whose coefficients were directly related to the outer scale, the inner scale, and the electron density fluctuations [*Knepp*, 1983b].

For the current experiment, the scattering model (1) was used to deduce information about the plasma density irregularities that cause L band scintillation. It enables (a) the identification of the ionospheric layers which cause scintillation and (b) the estimate of outer scale, correlation length, and axial ratio of the irregularities.

### 4.1 Identification of the Ionospheric Layers That Cause L Band Scintillation

Equation (1) can be reinterpreted in terms of the mean square total electron content (TEC) fluctuation along a particular raypath $\bar{{\left(\Delta N_{T}\right)}^{2}}$, avoiding assumptions on *L*_0_ and *D*, by invoking the autocorrelation function $B_{\Delta N_{T}}\left(\vec{\rho }\right)$ for Δ*N*_*T*_ [*Yeh and Liu*, 1982; *Forte*, 2008, 2012] as:

(2) $$\bar{{\left(\Delta \phi \right)}^{2}}=\lambda^{2}r_{e}^{2}B_{\Delta N_{T}}\left(0\right)=\lambda^{2}r_{e}^{2}\bar{{\left(\Delta N_{T}\right)}^{2}}$$

**Figure 6:**
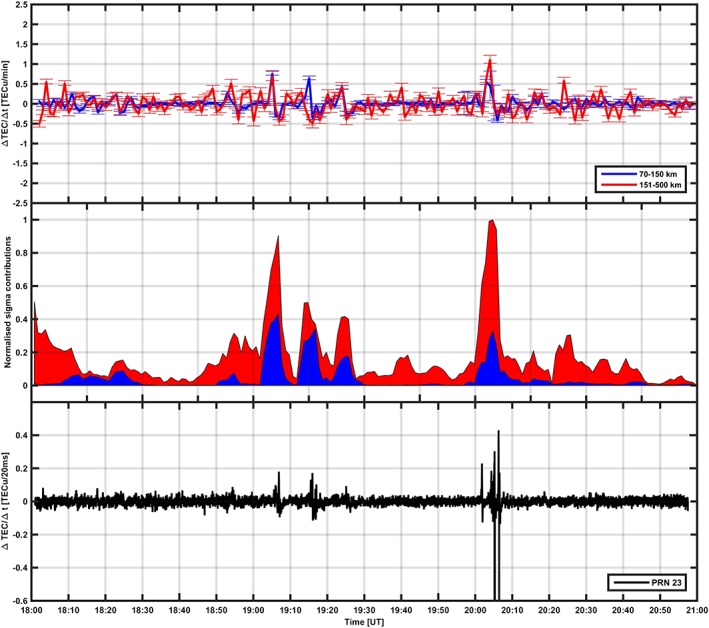
The event measured on 17 October 2013. (top) Temporal fluctuations in the radar TEC obtained integrating EISCAT electron density profiles for the *E* region (blue line) and the *F* region (red line), with error bars. (middle) The phase fluctuations originated from the *E* region (blue area) and the *F* region (red area). (bottom) The 50 Hz TEC temporal fluctuations on PRN32.

**Figure 7:**
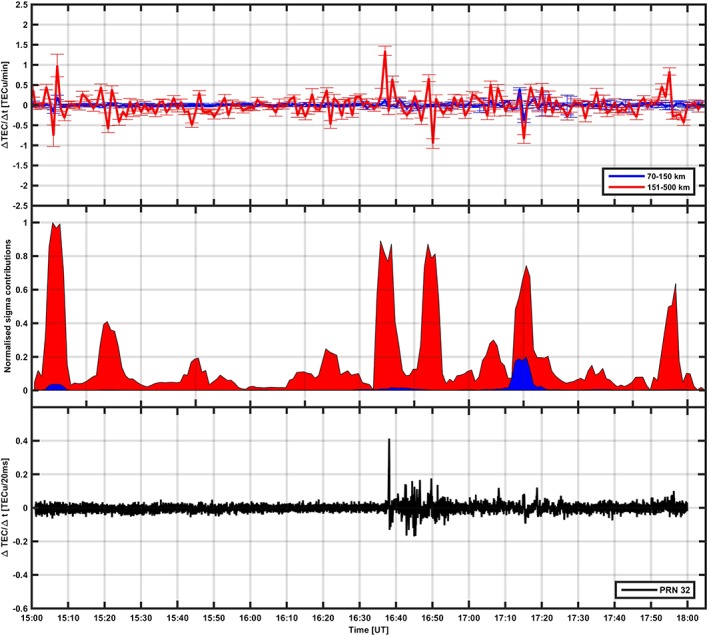
The event measured on 16 October 2013. (top) Temporal fluctuations in the radar TEC obtained integrating EISCAT electron density profiles for the *E* region (blue line) and the *F* region (red line), with error bars. (middle) The phase fluctuations originated from the *E* region (blue area) and the *F* region (red area). (bottom) The 50 Hz TEC temporal fluctuations on PRN23.

From the radar measurements $\bar{{\left(\Delta N_{T}\right)}^{2}}$ can be estimated by integrating electron density profiles over the *E* and *F* regions, respectively. Hence, the contribution to the phase fluctuations at L band corresponding to the *E* and *F* regions can be estimated according to (2). Figures 6 (top) and 7 (top) show temporal fluctuations of the radar electron density (i.e., Δ*N*_*T*_) profiles integrated over the *E* (blue) and *F* (red) region. $\bar{{\left(\Delta N_{T}\right)}^{2}}$ was used to estimate $\bar{{\left(\Delta \phi \right)}^{2}}$ from a given layer according to equation (2). The normalized contribution to the overall phase fluctuations from the *E* (blue area) and the *F* (red area) regions is shown in Figures 6 (middle) and 7 (middle). Because of coalignment, the GPS Δ*N*_*T*_ and phase scintillation index can be used to identify the origin of L band scintillation (bottom plot in Figures 6 and 7).

In the case of 17 October 2013, the radar TEC temporal fluctuations from the *E* region were larger and comparable to the fluctuations from the *F* region between 19:00–19:30 UT and 20:00–20:15 UT, with enhancements on the GPS phase scintillation index *σ*_*ϕ*_, on the low‐frequency phase PSD, and on the GPS TEC temporal fluctuations. This was consistent with the fact that Tromso was situated under the nightside of the auroral oval with auroral structures frequently traversing the radar beam (and the co‐aligned GPS raypath). The ionization in the *E* region was higher, and hence, the contribution to phase fluctuations from the *E* region was higher. In particular, between 20:00 and 20:15 UT larger phase fluctuations from both the *E* and *F* regions were consistent with particle precipitation occurring on the equatorward edge of the auroral oval at the onset of an auroral substorm (Figures 2 and 4).

The radar TEC temporal fluctuations and the 50 Hz GPS temporal fluctuations (Figure 6) indicate the source of phase fluctuations to be large‐scale ionization structures in the *E* and *F* regions during the periods 19:00–19:30 UT and 20:00–20:15 UT. After this, only structures in the *F* region have influence, despite the presence of a diffuse‐aurora sporadic *E* layer. A loss of lock on L2 semicodeless was observed between 20:00 and 20:15 UT (discontinuity on the GPS TEC temporal fluctuations).

The sporadic *E* layer (diffuse aurora) following between 20:15 and 21:00 on 17 October 2013 as well as the sporadic *E* layer noticed between 16:00 and 18:05 UT on 16 October 2013 did not produce any remarkable signature on TEC temporal fluctuations, phase PSDs, GPS carrier phase, or scintillation indices.

**Figure 8:**
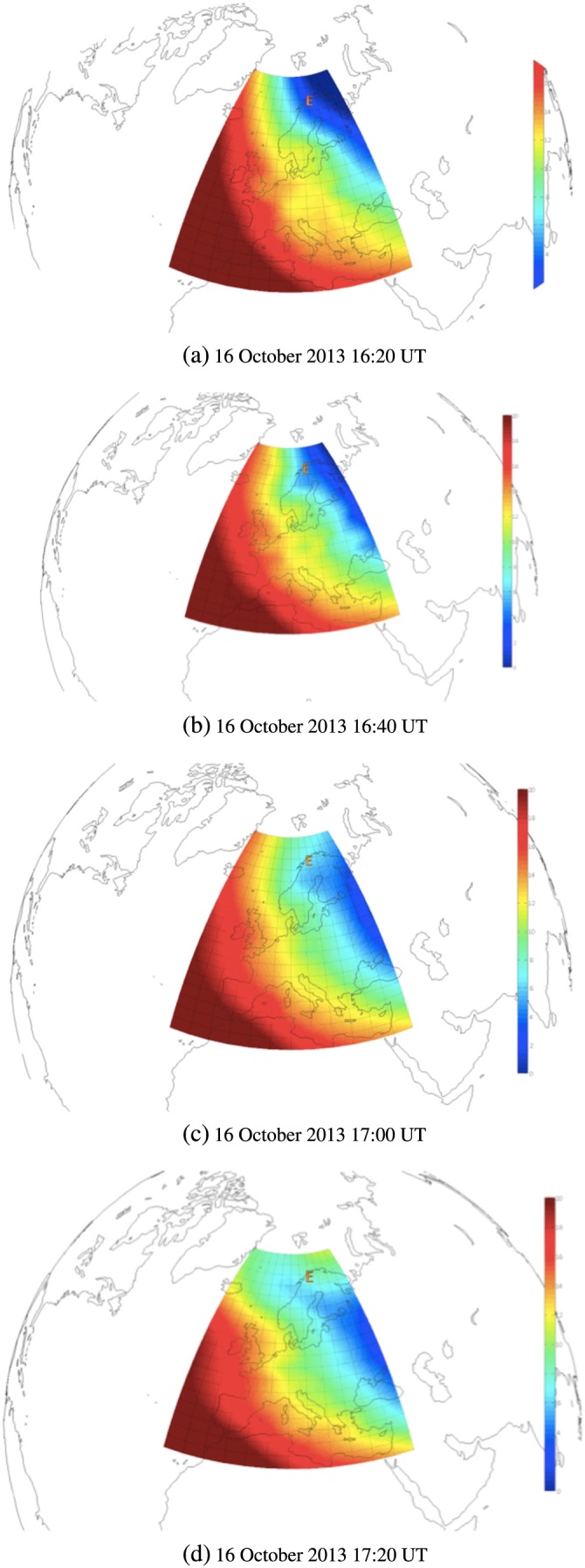
TEC maps reconstructed by tomographic imaging for 16 October 2013 between 16:20 and 17:20 UT. The trough forms south of Tromso between 16:40 and 17:20 UT. The location of the EISCAT radar close to Tromso is market as “E.” The trough can be recognized by the blue region indicating low TEC values extending across the EISCAT radar site between 16:40 and 17:20 UT.

In the case of 16 October 2013, the radar TEC temporal fluctuations between 16:30 and 17:00 UT were dominated by ionization structures in the *F* region; hence, phase fluctuations were mainly arising from the *F* region. Large‐scale structures in the *F* region caused enhancements on the GPS phase scintillation index *σ*_*ϕ*_, on the low‐frequency phase PSD, and on the GPS TEC temporal fluctuations. This is consistent with the presence of the trough across the radar beam between 16:30 and 17:00 UT indicated by a positive bay on the magnetic horizontal component (Figure 5) and further confirmed through TEC maps (Figure 8) [*Mitchell and Spencer*, 2003]. At around 17:15 UT another enhancement in the radar TEC temporal fluctuations with a larger contribution from the *E* region occurred without a marked signature on the GPS signals. This is consistent with the fact that after 17:00 UT, Tromso was situated under the evening side of the auroral oval where auroral arcs were frequently passing. Hence, ionization in the *E* region increased producing more contribution to phase perturbations arising from the *E* region. As opposed to the previous case, this ionization seemed to attain lower levels, and hence, it introduced smaller phase fluctuations overall. The radar TEC temporal fluctuations and the 50 Hz GPS TEC temporal fluctuations (Figure 7) indicate the origin of phase fluctuations from large‐scale ionization structures in the *F* region (16:30–17:00 UT), in both *E* and *F* regions (around 17:15 UT), and in the *F* region only afterward.

### 4.2 Estimate of Outer Scale, Correlation Length, and Axial Ratio

**Figure 9:**
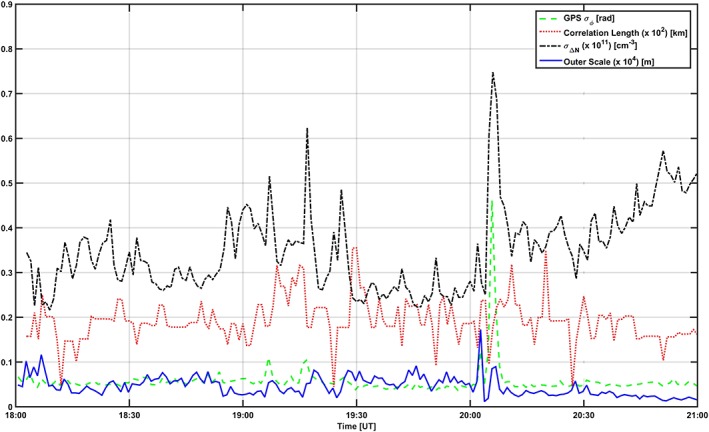
Estimate of propagation conditions on 17 October 2013: GPS phase scintillation index (green), standard deviation of electron density fluctuations along the line of sight (black), correlation length of ionization structures (red), and outer scale along the propagation direction (blue).

**Figure 10:**
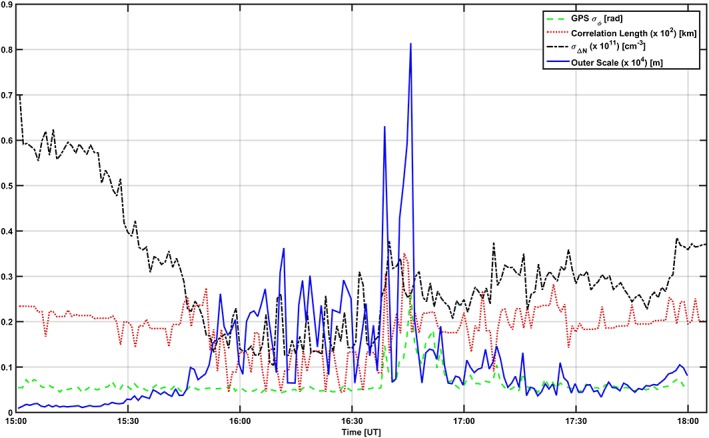
Estimate of propagation conditions on 16 October 2013: GPS phase scintillation index (green), standard deviation of electron density fluctuations along the line of sight (black), correlation length of ionization structures (red), and outer scale along the propagation direction (blue).

Assuming the description in (1), $\sigma_{\Delta N}=\sqrt{\bar{{\left(\frac{\Delta N}{N}\right)}^{2}}}$ can be estimated through the radar electron density profiles. Moreover, assuming that the phase scintillation index measured through the GPS L band signal is such that $\sigma_{\phi }^{2}=\bar{{\left(\Delta \phi \right)}^{2}}$ and that *D* sec *χ* ≈ 200 km (in view of the considerations above), then the outer scale along the propagation direction can be found by solving (1) for *L*_0_. Figures 9 and 10 show estimates of various quantities: the measured GPS phase scintillation index *σ*_*ϕ*_ (green), *σ*_Δ*N*_ (black), the correlation length of ionization structures (red) obtained from the radar electron density fluctuations, and *L*_0_ (blue).

The correlation length is confirmed to be always less than the assumed layer thickness, which justifies the use of a model such as (1) [*Booker and MajidiAhi*, 1981; *Knepp*, 1983a, 1983b]. The GPS phase scintillation increased with *σ*_Δ*N*_, as expected. However, the outer scale values varied in response to the combination between *σ*_Δ*N*_ and *σ*_*ϕ*_: enhancements in *σ*_*ϕ*_ corresponded to an outer scale of the order of 2 km (with a correlation length of the order of 20 km) in the case of 17 October 2013 and of the order of up to 8 km (with a correlation length of the order of 30 km) in the case of 16 October 2013. This indicates that the assumption of infinitely elongated irregularities along a direction transverse to propagation is more appropriate in the case of 16 October 2013 (16:30–17:00 UT) when *F* region large‐scale ionization was detected: the ionization showed more structure developed in the transverse plane, which seems to suggest axial ratios of the order of 10:10:1 for the trough. On the other hand, the assumption was less appropriate in the case of 16 October 2013 (after 17:00 UT) and 17 October 2013 (19:00–19:30 UT and 20:00–20:15 UT) when ionization due to particle precipitation was detected between the *E* and *F* regions: the ionization showed more structure field‐line elongated than in the transverse direction, with axial ratios of the order of 1:1:10.

In *Chartier et al*. [2016], the best fit to the observed scintillation required an axial ratio of 1, which indicates difficulties in modeling phase‐without‐amplitude scintillation at auroral latitudes.

## 5 Conclusions

The present analysis has identified and quantified for the first time a causal relationship between auroral ionization structures (as measured by the EISCAT UHF radar) and scintillation on GPS signals (as measured on GPS radio links co‐aligned with the radar beam). The EISCAT UHF radar was pointed in the line of sight of a given GPS satellite, the satellite signal being recorded by means of a GPS scintillation monitor co‐located with EISCAT UHF transmitter at Tromso.

The experiment has provided new insights into the type of structures that cause scintillation on GPS L band signals at auroral latitudes.

Large‐scale ionization structures extending between the *E* and *F* regions cause phase‐without‐amplitude scintillation on GPS L band signals, in response to low‐frequency enhancements on the PSD of the detrended GPS carrier phase. The absence of modifications on the PSD of the GPS intensity indicates for the first time that these large‐scale ionization structures did not cascade into smaller‐scale structures. Among all known mechanisms, the electron thermal diffusion instability [*Shalimov and Haldoupis*, 1995] seems possible in the auroral *E* region plasma but not capable of reproducing the observations.

The reason for observing phase scintillations without amplitude scintillations is due to an instability mechanism that injects free energy at the kilometer or larger scales, but does not allow for an inertial subrange energy cascade down to scales below the Fresnel scale, and to the use of a fixed cutoff frequency for detrending the GPS carrier phase [*Forte and Radicella*, 2002].

It remains to be understood exactly what physical mechanism is the cause of these observations: that is, a new instability mechanism that (a) operates in the *E* region of the ionosphere, (b) injects free energy at larger scales, and (c) does not allow for an energy cascade to smaller scales. Hence, more measurements and new theory are necessary to understand this issue.

In this experiment, large‐scale ionization structures were associated with the poleward edge of the ionospheric trough, with auroral arcs drifting across the radar beam on the nightside auroral oval and with particle precipitation at the onset of a substorm. The experiment indicated axial ratios of the order of 10:10:1 for *F* region irregularities associated with the trough and 1:1:10 for irregularities in the *E* and *F* region associated with particle precipitation.

Future experiments of the type described here will provide further information concerning the axial ratio of irregularities in the auroral ionosphere as well as on the structure function associated with them. These experiments will also provide more details on the mechanisms responsible for the inhibition of large‐to‐small‐scale cascade as well as on the modeling of propagation conditions leading to phase‐without‐amplitude scintillation at auroral latitudes. It is intended to include additional observations (Low‐Frequency Array, LOFAR, and Super Dual Auroral Radar Network, SuperDARN) in these future experiments in order to provide additional data concerning the structure of the ionospheric irregularities. In addition, the improvement of the model for phase‐without‐amplitude scintillation at auroral latitudes, following on *Chartier et al*. [2016], will be investigated.
